# Differences in the Association Between Modifiable Lifestyle Factors and Gastric Precancerous Lesions Among Mongolians and Han Chinese

**DOI:** 10.3389/fonc.2022.798829

**Published:** 2022-06-02

**Authors:** Weiwei Wang, Liying Qiao, Weiqi Dong, Jing Ren, Xiaotian Chang, Siyan Zhan, Peng Du, Yunfeng Xi, Shengfeng Wang

**Affiliations:** ^1^ National Clinical Research Center for Mental Disorders and Beijing Key Laboratory of Mental Disorders, Beijing Anding Hospital, Capital Medical University, Beijing, China; ^2^ The Inner Mongolia Autonomous Region Comprehensive Center for Disease Control and Prevention, Hohhot, China; ^3^ Baotou Medical College, School of Public Health, Baotou, China; ^4^ Department of Psychology, University of Michigan-Ann Arbor, Ann Arbor, MI, United States; ^5^ Department of Epidemiology and Biostatistics, School of Public Health, Peking University, Beijing, China; ^6^ Research Center of Clinical Epidemiology, Peking University Third Hospital, Beijing, China; ^7^ Center for Intelligent Public Health, Institute for Artificial Intelligence, Peking University, Beijing, China; ^8^ Key Laboratory of Carcinogenesis and Translational Research (Ministry of Education/Beijing), Department of Urology, Peking University Cancer Hospital and Institute, Beijing, China

**Keywords:** lifestyle factor, gastric precancerous lesions, ethnicity, disparity, Mongolians

## Abstract

**Background:**

There has been a paucity of evidence examining whether preventable behavioral risk factors led to ethnic differences of gastric precancerous lesions (GPL). We aimed to investigate the ethnic disparity of associations between GPL and lifestyle factors in Mongolian and Han Chinese populations.

**Methods:**

The study included participants aged 36-75 years enrolled in the Cancer Screening Program during 2016-2017 in Hohhot and Tongliao City, Inner Mongolia. GPL was defined as the gross cascading events (i.e., gastric ulcer, atrophic gastritis, intestinal metaplasia, and dysplasia) that preceded gastric cancer.

**Results:**

A total of 61638 participants were included, of whom 6863(11·1%) were Mongolians. Alcohol consumption was positively associated with GPL risk in both ethnic groups, but the magnitude was greater in Mongolians (odds ratio (OR) 6·91, 95%CI 5·82-8·28) than in Han Chinese (OR 5·64, 95%CI 5·27-6·04), corresponding to a higher population attributable fraction (PAF) for Mongolians (53·18% vs 43·71%). Besides, the strength of the positive association between physical inactivity and GPL risk was greater among Mongolians (OR 2·02, 95%CI 1·70-2·41; OR 1·09, 95%CI 1·02-1·17 among Han Chinese) with a higher PAF. Smoking was strongly associated with GPL risk in both ethnic groups as well, but the association was more prominent among Han Chinese (OR 5·24 (1·70-2·41) for <10 cigarettes/d, 8·19 (7·48-8·97) for 11-20 cigarettes/d, 7·07 (6·40-7·81) for ≥21 cigarettes/d; the corresponding ORs were 2·96 (2·19-4·00), 6·22 (5·04-7·68), and 7·03 (5·45-9·08) among Mongolians). Lastly, our findings revealed that a significant correlation between insufficient fruits and vegetable consumption and GPL risk was only found among Mongolians (OR 1·27, 95%CI 1·04-1·56).

**Conclusions:**

Our result suggested that high-risk lifestyle factors should be reduced, particularly in Mongolians. Further studies are needed to elucidate the underlying mechanisms and to reduce health disparities in underserved ethnic groups.

## Introduction

Gastric cancer (GC) is the fifth most common cancer worldwide (1). With more than one million new cases globally, GC is one of the leading causes of cancer death, resulting in approximately 800,000 deaths in 2018 alone (1). More than 60% of all GC cases were observed in Eastern and South-Eastern Asia in 2018, making it the region with the highest incidence of GC (2). Specifically, GC has become a rising public health concern in China. Over the past two decades, China has witnessed a substantial rise in GC incidence rates, disease burdens, and mortality rates (3). Invasive gastric carcinoma is often preceded by a series of degrading conditions known as gastric precancerous lesion (GPL), including gastric ulcers (GU), atrophic gastritis (AG), intestinal metaplasia (IM), and dysplasia. The timely detection and treatment of these conditions would reduce GC incidence significantly and increase survival rates.

A previous study has identified some modifiable lifestyle factors as potential contributors to cancer development (4). One meta-analysis and prospective cohort study found an association between increased GC rate and unhealthy lifestyle factors (i.e., smoking, alcohol consumption, preserved food consumption, infrequent intake of fresh fruit and vegetables) (5). A significant inverse association was observed between the number of healthy lifestyle factors and the incidence of GC even when genetic risk was also adjusted for, demonstrating the prominent impact of lifestyle factors on GC (5).

The incidence of GC varies across geographical regions and ethnic groups. According to the GLOBOCAN 2018 study, GC incidence is highest among Eastern Asians (32·1 for men and 13·2 for women per 100,000) and lowest among Northern Africans (4·7 for men and 3·0 for women per 100,000) (1). Likewise, disparities in GC incidence are also observed among different ethnic groups within a country, e.g. non-Hispanic Black Americans have an almost two-fold higher overall incidence rate than non-Hispanic White Americans (6). Despite widespread consensus that the incidence of GC/GPL differs, no comprehensive explanatory model for this has yet been proposed. Genetic differences between populations may account for some of these differences, but some researchers believe lifestyles adopted by the different populations may also have a different impact (7, 8). Nevertheless, most current studies examining the relationship between lifestyle factors and GC/GPL focus primarily on differences within wider geographical regions (continents, countries) (7, 8). In China, particularly, the majority of studies regarding GC/GPL either were indiscriminating about ethnicities or included only Han Chinese patients (5).

To address this gap, our cross-sectional study will investigate the ethnic differences of modifiable lifestyle factors and GPL risks among Mongolian Chinese and Han Chinese. Based on their different cultural heritage and lifestyles, we hypothesized that the two groups would have significantly different risks of developing GPL. This study would expand our understanding of GC/GPL among the Chinese Mongolian population, who are consistently underrepresented in research in this area.

## Materials and Methods

### Study Population

The Cancer Screening Program in Urban China (CanSPUC), a national cancer screening program, was conducted *via* a two-stage sampling between 2016 and 2017. At the primary stage, we selected the provincial capital Hohhot and Tongliao (with 31·55% Mongolian residents in the urban area), the two cities with the highest proportion of Mongolian residents in Inner Mongolia. At the secondary stage, we selected community healthcare centers with adequate research foundations and consent to participate under the jurisdiction of each urban area. Finally, five (Hainban, Dongfeng east, Zhaowuda, University west, and Wulan east community healthcare centers) and three (Hongxing, Huolin, and Shijie community healthcare centers) community healthcare centers under the jurisdiction of Hohhot city and Tongliao city respectively were included. Extensive publicity campaigns were launched and invitation letters (with study information leaflets) were delivered door-to-door by local community leaders or health workers afterwards. Potential participant were approached by trained staff by means of phone calls or visits (9). All participants were required to bring their unique national identity (ID) cards to the assessment center set up in the local community. All individuals aged 35-74 years who had lived in the local district for at least three years without a major disability and previous cancer diagnoses were recruited voluntarily for the baseline survey from the eight community healthcare centers. Community healthcare centers, at the bottom three-tiered health care delivery system, mainly provide such basic medical and public health services as disease prevention and control, healthcare, health education, disease management, creation of residents health records, diagnosis and treatment of common or frequently-occurring diseases, and rehabilitation and nursing of patients suffering from certain diseases, accepting patients referred by hospitals, and referring patients beyond their service capacity to hospitals (10, 11).

Each eligible participant completed an interviewer-administered Cancer Risk Assessment Questionnaire that consisted of seven major sections related to general demographics, dietary habits, lifestyle behaviors, mental status, personal and family medical histories, physical measurements (i.e. height, weight), and reproductive history (for women) (12). Ethics approvals were obtained from the Ethics Committee of National Cancer Center/Cancer Hospital. All participants provided a written informed consent form.

A total of 70,010 participants attended the baseline survey. The response rates of Mongolians and Han Chinese were 30.63% (7925/25875) and 52.30% (60335/115369) respectively. We excluded 8372 (11·96%) participants with any other upper gastrointestinal disease and who were not Han Chinese or Mongolian, yielding an overall of 61,638 participants included in the present analysis.

### Assessment of Behavioral Risk Factors

Individuals were classified as never, former, or current smokers based on their smoking status. Current smokers were defined as those who had smoked ≥ 1 cigarette (or equivalent) per day for at least 6 months at baseline. Former smoker was defined as smoking ≥ 1 cigarette (or equivalent) per day for more than 6 months but had quit smoking by choice for ≥ 6 months before baseline. Participants classified as current and past smokers were further inquired about the number of cigarettes, duration (year) of smoking, and duration (year) of cessation. The number of cigarettes consumed per day was categorized into light (≤10 cigarettes), medium (11-20 cigarettes), and heavy (≥21 cigarettes). Participants were classified into three categories: never, former, and current drinkers. Current drinkers were those who had consumed alcohol at least once a week on average for more than 6 consecutive months. Former drinker was defined as consuming alcohol at least once a week on average for more than 6 consecutive months but had quit drinking by choice for ≥ 6 months before baseline. Regular exercise was defined as an average of three or more days a week for at least 30 minutes each (13). Participants were divided into two groups, physically active and physically inactive, based on this criterion. Consisting with the WHO recommendation of consuming five portions (400g) or more of fruit and vegetables daily (14), this study defined insufficient fruit and vegetable intake as consuming less than 360g vegetables and 180g fruits per day on average.

### Assessment of Covariates

Covariate information was obtained from the baseline questionnaire covering age (years), sex, education (primary school and lower, junior school, high school, college and higher), occupation (public officer, agricultural and industrial service personnel, house worker, others), and family history of gastric cancer (yes/no). Weight and height were measured using calibrated instruments by trained personnel. Body mass index (BMI) was calculated by dividing weight in kilograms by height in meters squared.

### Ascertainment of Outcomes

The primary outcome, the prevalence of GPL, was defined as a self-reported diagnosis of ‘gastric ulcer’, ‘atrophic gastritis’, ‘intestinal metaplasia’ or ‘dysplasia’ given by a secondary or tertiary-level hospital (15).

### Statistical Analyses

We constructed a lifestyle risk factor index by summing the individual scores of four risk factors (1=yes, 0=no): smoking, drinking, physical inactive, and insufficient intake of vegetables and fruits (16). Former smoking and current smoking were combined as smoking, and former drinking and current drinking were combined as drinking. The Student’s t test and the chi2 test for categorical variables were used to identify basic differences between ethnicities. In the analysis of individual lifestyle factors, separate logistic regression models were used to estimate adjusted odds ratios (ORs) and 95% confidence intervals (CIs) for GPL in Han Chinese and Mongolians. Model 1 was adjusted for age and sex. Model 2 included the variables adjusted for in Model 1, education and occupation. Model 3 included all variables adjusted for in Model 2, and the following variables of BMI (<18·5, 18·5 to 24, 24 to 28, ≥28) and family history of gastric cancer. The same adjustment was made in the analysis of combined lifestyle factors. Potential confounders included as covariates in multivariable models were selected according to a previous understanding of risk factors for GPL. Interactions with ethnicities were examined by including appropriate interaction terms in the logistic regression models. We conducted sensitivity analysis with ruling out participants with family history of gastric history.

Population attributable fractions (PAFs) represent the proportion of GPL that is attributable to unfavorable exposure to behavioral risk factors. PAFs are calculated based on the adjusted OR of GPL and the prevalence of exposure to the behavioral risk factors in the total population (P) using the following formula (17):


$$PAF=\frac{P(OR-1)}{P(OR-1)+1}X100\%$$

The confidence interval of PAF was quantified with the simulation technique incorporating sources of uncertainty of OR and exposure prevalence estimates obtained from our population.

All statistical analyses were performed using Stata^®^ version 16·0 (StataCorp. LLC, College Station, TX 77845, USA). All P-values were 2-sided, and the significance level was set at P < 0·05.

### Data Availability

Deidentified participant data will be available through reasonable request to the corresponding authors.

### Role of the Funding Source

The funders had no role in the study design, data collection, data analysis, data interpretation, or writing of the report. The corresponding authors had full access to all data in the study and had final responsibility for the decision to submit for publication.

## Results

### Baseline Characteristics

This study enrolled 61638 participants, including 54775 (88·87%) Han Chinese and 6863 (11·13%) Mongolians. The mean (SD) age of Han and Mongolians was 55·81 (9·03) years and 54·29 (8·65) years, respectively. And the percentage of female participants was higher for Mongolians (60·03%) than Han Chinese (51·8%). Compared with Han participants, Mongolians had substantially higher BMI and education level and were more likely to have GC family history (**Table 1**).

**Table 1:** Basic characteristics of participants grouped by ethnicity.

Characteristics	Total, N=61638	Han, n=54775	Mongolians, n=6863
Women	32492 (52.71)	28372 (51.80)	4120 (60.03)
Age, mean (SD)	55.63 (9.03)	55.81 (9.03)	54.29 (8.65)
Body mass index (kg/m^2^), n (%)^*^			
<18.5	1095 (1.78)	992 (1.81)	103 (1.50)
18.5-23.9	30625 (49.69)	27618 (50.42)	3007 (43.81)
24-27.9	25166 (40.83)	22172 (40.48)	2994 (43.63)
≥28	4427 (7.18)	3711 (6.77)	716 (10.43)
Education, n (%)			
College and higher	11002 (17.85)	9130 (16.67)	1872(27.28)
High school	17886 (29.02)	15782 (28.81)	2104 (30.66)
Junior school	20014 (32.47)	18193 (33.21)	1821 (26.53)
Primary school and lower	12736 (20.66)	11670 (21.31)	1066 (15.53)
In marriage at time of enrollment	59991 (97.33)	53357 (97.41)	6634 (96.66)
Occupation, n (%)			
Public officer	20399 (33.09)	18061 (32.97)	2338 (34.07)
Agricultural and industrial service personnel	29304 (47.54)	26198 (47.83)	3106 (45.25)
House worker	9445 (15.32)	8428 (15.39)	1017 (14.82)
Others	2490 (4.04)	2088 (3.81)	402 (5.86)
Has family history of gastric cancer	2352 (3.82)	1987 (3.63)	365 (5.32)
Smoking, n (%)^†^			
Non-smoker	48307 (78.37)	43240 (78.94)	5067 (73.83)
Current smoker	11881 (19.28)	10296 (18.80)	1585 (23.09)
Former smoker	1450 (2.35)	1239 (2.26)	211 (3.08)
Number of cigarettes smoked per day, Median (P_25_, P_75_)^‡^	20 (10, 18)	20 (10, 18)	20 (10, 18)
Duration of smoking, Median (P_25_, P_75_) (year)^§^	25 (19, 20)	25 (19, 20)	27 (19, 21)
Alcohol consumption, n(%)^¶^			
Non-drinker	48579 (78.81)	43603 (79.60)	4976 (72.51)
Current drinker	12034 (19.52)	10314 (18.83)	1720 (25.06)
Former drinker	1025 (1.66)	858 (1.57)	167 (2.43)
Physically inactive, n (%)^#^	38253 (62.06)	34125 (62.30)	4128 (60.15)
Insufficient vegetables and fruits intake, n (%)^||^	50569 (82.04)	45039 (82.23)	5530 (80.58)

^*^325 (0.53%) missing. ^†^Current smokers were defined as those who had smoked ≥ 1 cigarette (or equivalent) per day for at least 6 months at baseline. ^‡^158 (0.26%) missing. ^§^587 (0.86%) missing. ^¶^Alcohol consumption was defined as drinking alcohol at least once a week on average for more than 6 consecutive months. ^#^Regular physical activity was defined as exercise at least ≥30 minutes on 3 days of the week. ^||^Insufficient vegetables and fruits intake was defined as consuming less than 360g vegetables and 180g fruits per day on average.

Of the overall population, 28·87%, 45·44%, 9·85%, and 8·40% had one, two, three, and four high-risk lifestyle factors, respectively (**Supplementary Table 1**). Mongol participants were more likely to be current drinkers, current smokers, and have a longer duration of smoking relative to Han participants. Prevalence of insufficient physical activity and fruits and vegetable intake were similar across the two ethnic groups, with the rate in Mongolians being slightly lower (all P<0·05) (**Table 1**). In total, 6509 (10·56%) participants reported GPL. Specifically, 5580 (10·19%) Han Chinese participants and 929 (13·54%) Mongolian participants reported a diagnosis of GPL.

### Association of Lifestyle Factors With GPL

In univariate analysis, smoking, alcohol consumption, physical inactivity, and having three or more high-risk lifestyle factors were associated with an elevated risk of GPL in both Han and Mongolian participants, while insufficient intake of vegetables and fruits was significantly associated with GPL risk only in Mongolians (all P<0·05) (**Table 2**). After adjusting for covariates, including age, sex, education, occupation, BMI, and family history of gastric cancer, the associations were partially attenuated but remained statistically significant (**Table 3**). For Mongolians, compared with never smokers, the multivariable-adjusted ORs were 2·96 (95% CI: 2·19-4·00), 6·22 (95% CI: 5·04-7·68), and 7·03 (95% CI:5·45-9·08) for those who smoked <10, 711-20, and ≥21 cigarettes per day, respectively. The corresponding ORs among Chinese Hans were 5·24 (95% CI: 4·66-5·90), 8·19 (95% CI: 7·48-8·97), and 7·07 (95% CI: 6·40-7·81), indicating a significant interaction between smoking and ethnicity for GPL (P=0·009). Compared with normal weight, underweight, overweight, and obesity were associated with higher risk of GPL for Han Chinese, while only obesity was significantly associated with GPL risk for Mongolians. Compared with public officer, agricultural and industrial service personnel had elevated risk of GPL in both Han Chinese and Mongolians (**Supplementary Table 2**).

**Table 2:** Univariate analysis of the number of behavioral risk factors for GPLs by ethnic group.

Risk factors	Han				Mongolians				*P*-value^*^
	control, n(%)	patients with GPLs, n(%)	OR (95% CI)		control, n(%)	patients with GPLs, n(%)		OR (95% CI)	
Overall	49195 (100.00)	5580 (100.00)	–		5934 (100.00)	929 (100.00)	–		–
Smoking (cigarettes/d) ^†^									0.040
Never	40980 (83.30)	2260 (40.50)	Ref		4697 (79.15)	370 (39.83)	Ref		
<11	2173 (4.42)	497 (8.91)	4.15 (3.73,4.61)		325 (5.48)	79 (8.50)	3.09 (2.36,4.03)		
11-20	3012 (6.12)	1262 (22.62)	7.60 (7.03,8.22)		499 (8.41)	250 (26.91)	6.36 (5.29,7.36)		
≥21	2045 (4.16)	1307 (23.42)	11.59 (10.68,12.57)		254 (4.28)	178 (19.16)	8.90 (7.15,11.07)		
Alcohol consumption^‡^	7351 (14.94)	2964 (53.12)	6.45 (6.09,6.84)		1143 (19.26)	577 (62.11)	6.87 (5.93,7.96)		0.434
Physically inactive^§^	30369 (61.73)	3756 (67.31)	1.28 (1.20,1.35)		3414 (57.53)	714 (76.86)	2.45 (2.09,2.88)		<0.001
Insufficient vegetables and fruits intake^¶^	40451 (82.23)	4588 (82.22)	1.00 (0.93,1.07)		4747 (80.00)	783 (84.28)	1.34 (1.11,1.62)		0.004
Lifestyle risk factor index^#^									<0.001
0	3726 (7.57)	283 (5.07)	Ref		532 (8.97)	41 (4.41)	Ref		
1	14932 (30.35)	853 (15.29)	0.75 (0.65,0.86)		1908 (32.15)	105 (11.30)	0.71 (0.49,1.04)		
2	23850 (48.48)	1500 (26.88)	0.83 (0.73,0.94)		2430 (40.95)	229 (24.65)	1.22 (0.87,1.73)		
3	3979 (8.09)	1255 (22.49)	4.15 (3.62,4.76)		642 (10.82)	198 (21.31)	4.00 (2.80,5.71)		
4	2708 (5.50)	1689 (30.27)	8.21 (7.17,9.40)		422 (7.11)	356 (38.32)	10.95 (7.73,15.50)		

GPL, gastric precancerous lesions; OR, odds ratio; CI, confidence interval. ^*^P-value of interaction between Mongolians and Han. ^†^158 (0.26%) missing. Smokers were defined as those who had smoked ≥ 1 cigarette (or equivalent) per day for at least 6 months. ^‡^Alcohol consumption was defined as drinking alcohol at least once a week on average for more than 6 consecutive months. ^§^Regular physical activity was defined as exercise at least ≥30 minutes on 3 days of the week. ^¶^Insufficient vegetables and fruits intake was defined as consuming less than 360g vegetables and 180g fruits per day on average. ^#^Lifestyle risk factor index was calculated by summing the individual scores of four risk factors (1=yes, 0=no): smoking, drinking, physically inactive, and insufficient intake of vegetables and fruits.

**Table 3:** Multivariable-adjusted ORs(95%CIs) for GPL by lifestyle factors* grouped by ethnicity.

	Model 1 (Age, Sex)		Model 2 (Age, Sex, Education, Occupation)		Model 3 (Age, Sex, Education, Occupation,BMI^†^, Family history of gastric cancer)	
	OR (95%CI)	*P*-value^‡^	OR (95%CI)	*P*-value^‡^	OR (95%CI)	*P*-value^‡^
**Smoking^§^**		0.043		0.031		0.009
Han						
Never	Ref		Ref		Ref	
<11 cigarettes/d	5.53 (4.95, 6.17)		5.33 (4.77,5.95)		5.24 (4.66,5.90)	
11-20 cigarettes/d	9.96 (9.17,10.82)		9.46 (8.70,10.29)		8.19 (7.48,8.97)	
≥21 cigarettes/d	13.43 (12.35, 14.61)		12.36 (11.53,13.46)		7.07 (6.40,7.81)	
Mongolians						
Never	Ref		Ref		Ref	
<11 cigarettes/d	3.73 (2.84,4.91)		3.54 (2.69,4.68)		2.96 (2.19,4.00)	
11-20 cigarettes/d	7.62 (6.28,9.25)		7.25 (5.96,8.82)		6.22 (5.04,7.68)	
≥21 cigarettes/d	10.50 (8.37,13.17)		10.28 (8.17.12.93)		7.03 (5.45,9.08)	
**Alcohol consumption**		0.250		0.212		0.015
Han	7.87 (7.40,8.37)		7.53 (7.08,8.01)		5.64 (5.27,6.04)	
Mongolians	8.63 (7.36,10.12)		8.32 (7.09,9.77)		6.91 (5.82,8.28)	
**Physically inactive**		<0.001		<0.001		<0.001
Han	1.28 (1.20,1.36)		1.21 (1.14,1.28)		1.09 (1.02,1.17)	
Mongolians	2.46 (2.09,2.89)		2.35 (1.99,2.77)		2.02 (1.70,2.41)	
**Insufficient vegetables and fruits intake**		0.003		0.003		0.060
Han	1.00 (0.93,1.08)		0.96 (0.89,1.03)		1.04 (0.96,1.13)	
Mongolians	1.35 (1.12,1.63)		1.30 (1.07,1.57)		1.27 (1.04,1.56)	
**Lifestyle risk factor index^¶^**		<0.001		<0.001		0.007
Han						
0	Ref		Ref		Ref	
1	0.78 (0.68,0.90)		0.77 (0.67,0.89)		0.81 (0.70,0.94)	
2	0.89 (0.78,1.01)		0.84 (0.74,0.96)		0.91 (0.79,1.05)	
3	5.08 (4.42,5.83)		4.69 (4.08,5.39)		3.81 (3.28,4.42)	
4	10.26 (8.94,11.78)		9.22 (8.03,10.60)		6.97 (6.00,8.09)	
Mongolians						
0	Ref		Ref		Ref	
1	0.74 (0.51,1.07)		0.72 (0.50,1.05)		0.75 (0.51,1.10)	
2	1.31 (0.93,1.85)		1.28 (0.90,1.81)		1.22 (0.85,1.75)	
3	4.82 (3.36,6.91)		4.65 (3.23,6.69)		3.83 (2.62,5.59)	
4	13.89 (9.73,19.83)		12.94 (9.03,18.54)		9.79 (6.74,14.21)	

OR, odds ratio; CI, confidence interval.*Smokers were defined as those who had smoked ≥ 1 cigarette (or equivalent) per day for at least 6 months. Alcohol consumption was defined as drinking alcohol at least once a week on average for more than 6 consecutive months. Regular physical activity was defined as exercise at least ≥30 minutes on 3 days of the week. Insufficient vegetables and fruits intake was defined as consuming less than 360g vegetables and 180g fruits per day on average. §158 (0.26%) missing. ‡P-value of interaction between Mongolians and Han. †325 (0.53%) missing. ¶Lifestyle risk factor index was calculated by summing the individual scores of four risk factors (1=yes, 0=no): smoking, drinking, physically inactive, and insufficient intake of vegetables and fruits.

Compared with nondrinkers, both Mongolian (OR = 6·91, 95% CI: 5·82-8·28) and Han Chinese (OR = 5·64, 95% CI: 5·27-6·04) drinkers were at higher risk for GPL (P=0·015 for interaction with ethnicity). In addition, physical inactivity was associated with a two-fold increase in GPL risk among Mongolians (OR=2·02, 95% CI: 1·70-2·41)· However, this relationship was less prominent among Han Chinese (OR=1·09, 95% CI: 1·02-1·17; P<0·001 for interaction with ethnicity). Furthermore, the association between insufficient vegetables and fruits intake and GPL was only observed in Mongolians (OR=1·30, 95% CI: 1·07-1·57), but the P-value for interaction with ethnicity was not significant (P=0·060).

For combined lifestyle factors, no association was found between one or two behavioral risk factors and GPL in either Han or Mongolian participants. Among Mongolians, compared with participants without any lifestyle risk factors, the adjusted ORs were 3·83 (96%CI: 2·62-5·59) and 9·79 (95%CI 6·74-14·21) for participants with three and four lifestyle risk factors, respectively. The corresponding ORs were 3·81 (95% CI: 3·28-4·42) and 6·97 (95% CI:6·00-8·09) among Han participants.

**Figure 1:**
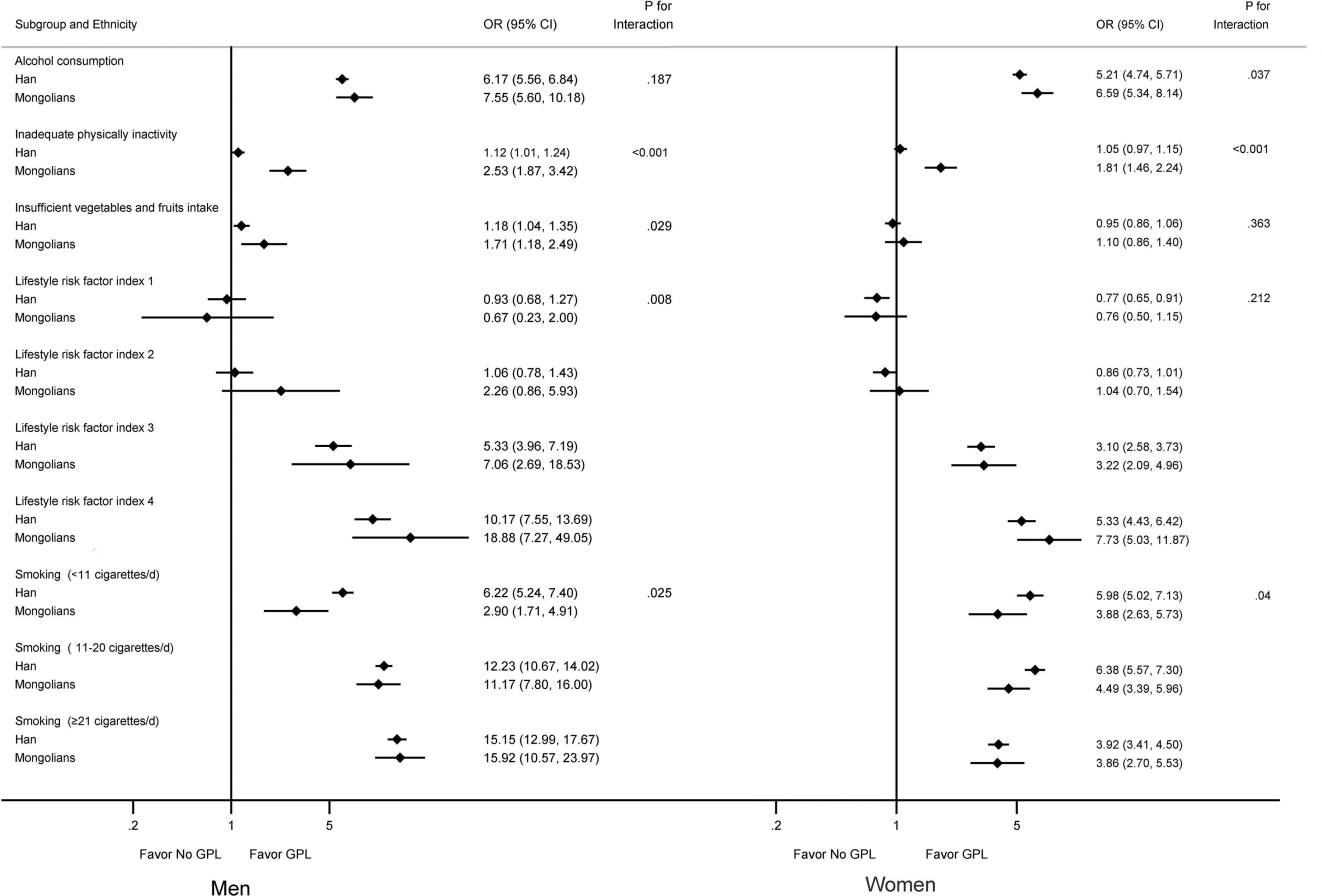
Multivariable-adjusted odds ratios for GPL by lifestyle factors grouped by sex and ethnicity. Smokers were defined as those who had smoked ≥ 1 cigarette (or equivalent) per day for at least 6 months. Alcohol consumption was defined as drinking alcohol at least once a week on average for more than 6 consecutive months. Model was adjusted for age, education, occupation, body mass index, and family history of gastric cancer. P-value of interaction between sex and ethnicity. OR, odds ratio. CI, confidence interval. GPL, gastric precancerous lesions.

All associations of lifestyle factors with the risk of GPL differed by ethnicity (P_interaction_ < 0.05), except for alcohol consumption in men (P_interaction_ = 0.187) and insufficient intake of vegetables and fruits (P_interaction_ = 0.363) and lifestyle risk factor index (P_interaction_ = 0.212) in women (**Figure 1**).

Results of our sensitivity analysis were substantially consistent with primary findings (**Supplementary table 3**). To estimate the comprehensive effect of smoking and alcohol on GPL, we constructed an index by combining smoking with drinking and found that compared with the No smoking & No drinking group, the risk for GPL of the Smoking & Drinking group increased significantly across both sexes and both ethnic groups, as well as the Smoking or Drinking group (**Supplementary Table 4**).

### Population Attributable Fractions

Among Mongolians participants, alcohol consumption had the largest PAF (53·18%, 95%CI 49·02%-57·00%) for GPL, followed closely by smoking (43·38%, 95%CI 39·09%-47·37%), physical inactivity (38·79%, 95%CI 30·45%-46·13%), and insufficient intake of vegetables and fruits (18·07%, 95%CI 2·54%-31·12%) (**Table 4**). However, smoking was the risk factor with the highest PAF (46·25%, 95%CI 44·63%-47·82%) among Chinese Hans. If participants modified any of the lifestyle factors, an estimated 44·40% (95%CI 21·30%-60·72%) and 31·18% (95%CI 20·88%-40·01%) of GPL would be avoided among the Mongolians and the Chinese Hans, respectively. If participants changed all four lifestyle factors, the numbers of GPL in Mongolia and Chinese Hans would be reduced by 32·77% (95%CI 29·19%-36·17%), and 25·13% (95%CI 23·78%-26·46%), respectively.

**Table 4:** The fraction of gastric precancerous cancer attributable (PAF) to lifestyle factors* grouped by ethnicity.

	Number at risk	Cases, n (%)	PAF (95%CI) (%)^†^
Smoking			
Han	11535	3320 (28.78)	46.25 (44.63,47.82)
Mongolians	1796	559 (31.12)	43.38 (39.09,47.37)
Alcohol consumption			
Han	10315	2964 (28.73)	43.71 (42.06,45.31)
Mongolians	1720	577 (33.55)	53.18 (49.02,57.00)
Physically inactive			
Han	34125	3756 (11.01)	5.64 (1.29,9.79)
Mongolians	4128	714 (17.30)	38.79 (30.45,46.13)
Insufficient vegetables and fruits intake			
Han	45039	4588 (10.19)	3.00 (-3.91,9.46)
Mongolians	5530	783 (14.16)	18.07 (2.54,31.12)
Having four risk factors			
Han	4397	1689 (38.41)	25.13 (23.78,26.46)
Mongolians	778	356 (45.76)	32.77 (29.19,36.17)
Having any risk factors			
Han	50875	5312 (10.44)	31.18 (20.88,40.01)
Mongolians	6308	890 (14.11)	44.40 (21.30,60.72)

OR, odds ratio; CI, confidence interval. ^*^Smokers were defined as those who had smoked ≥ 1 cigarette (or equivalent) per day for at least 6 months. Alcohol consumption was defined as drinking alcohol at least once a week on average for more than 6 consecutive months. Regular physical activity was defined as exercise at least ≥30 minutes on 3 days of the week. Insufficient vegetables and fruits intake was defined as consuming less than 360g vegetables and 180g fruits per day on average. ^†^Population attributable fraction was adjusted for age, sex, education, occupation, body mass index and family history, and there are 325 (0.53%) missing values in body mass index.

## Discussion

This study aimed to investigate the differences in the associations between GPL risk and lifestyle factors among Mongolian and Han Chinese living in China mainland. We have identified significant ethnic differences in the relationship between GPL and modifiable lifestyle factors. Alcohol consumption and inadequate physical activity were more strongly associated with GPL development among Mongolians, whereas the association between smoking and GPL development was more prominent among Han Chinese. We also found that insufficient vegetable and fruit consumption elevated the risk of GPL for Mongolians, but not for Han Chinese.

(1) Compared with Han Chinese, Mongolians had a significantly higher proportion of alcohol consumption (23·1% vs 18·8%). The association between alcohol consumption and GPL development was also stronger for Mongolians (6·91 vs 5·64) and a larger percentage of the Mongolian population was attributable to alcohol consumption (PAF 53·18% vs 43·71%). Such differences might reflect genetic and biological differences among the two groups.

First, ALDH2*G homozygote, a genetic variation associated with heavy drinking, has been found to be more prevalent among Mongolians than Han Chinese (22). A population based study from Japan also showed that individuals with ALDH2 typical homozygote consumed significantly larger quantities of ethanol than those with ALDH2 heterozygote (23). Indeed, the possible genetic predisposition corresponds to Mongolians’ drinking patterns. In a cross-sectional survey on alcohol usage, 30·12% (772/2563) of Mongolians satisfied the criteria of heavy drinkers (over 25 g/day of alcohol) (19). In addition, the China National health survey (CNHS) showed that the lifetime prevalence of alcohol usage was higher among Mongolians than Han Chinese (50·27% vs 40·76%, P<0·001). Excessive alcohol consumption among Mongolians thus results in an increased risk of GI cancer, as studies have demonstrated the relationship between alcohol use and GI cancer rates (24).

Second, aside from directly affecting the risk of GI cancer, alcohol consumption also impairs the efficacy of Helicobacter pylori treatment. A study had shown that compared to alcohol non-consumers, failures to eradicate H· pylori occurred significantly more often among alcohol consumers (OR 4·4) (25), and H· pylori infection is one of the strongest predictors for gastric cancer. The compromising effect of alcohol on HP treatment is partially reflected in the prevalence of HP infection among these two ethnic groups. While a cross-sectional study reveals a 40·8% prevalence rate of HP infection among Han Chinese (26), another research comparing Mongolian and Japanese populations indicated that the prevalence rate of HP among Mongolians was as high as 76% (27). Compounded with their genetic determinants of resistance to several antibiotics (i.e., metronidazole and levofloxacin) commonly used for HP treatment (18), Mongolians’ excessive alcohol consumption further enervates the treatment efficacy for HP, leaving them at heightened risk of GI cancer.

Third, heavy alcohol consumption might induce changes in gene expression among the Mongolian population. One study had identified that the cytochrome P4502E1 (CYP2E1) allele frequencies of Mongolian subjects differed from those of other ethnic groups in East Asia (i.e., Koreans, Japanese, and Han-Chinese) (28). This genetic variation might result from alcohol usage, as one research found that heavy (40 g/day) intake of alcohol markedly induces expression of CYP2E1 in the gastrointestinal mucosa of rodents and humans (29). The abnormal expression of CYP2E1 contributes to the formation of reactive oxygen species in the gastrointestinal tract and the activation of procarcinogens such as nitrosamines, which further elevates the risk of gastric cancer (29).

(2) Mongolians were less likely to be physically inactive than Han Chinese (60·1% vs 62·3%) but had a significantly stronger association for inadequate physical activity with GPL and a higher PAF (P<0·05).

First, Mongolian people had been found to have higher obesity rates, higher blood pressure, and lower high-density lipoprotein cholesterol levels than Han Chinese. These factors are all indicators of poor metabolic health, which, according to the Framingham Heart Study result (30), put individuals at a higher risk of cancer. One potential explanation for this phenomenon is that metabolically unhealthy individuals had the propensity to develop more local ectopic fat, which contributes to systematic inflammation and serves as a favorable environment for tumor development (31).

Second, inadequate physical activity impairs glucose metabolism (20). Glucose metabolism impairment was more prevalent in Mongolians with an impaired fasting glucose rate of 18·5% compared with 7·3% in overall Chinese adults (32), resulting in increased glycated hemoglobin levels. An elevated glycated hemoglobin level is an indicator of diabetes, which leads to an increase in reactive oxygen species and oxidative damage (21). These changes are known to contribute to the development of gastric cancer by causing mutations in oncogenes and tumor suppressor genes (33).

(3) Furthermore, although Mongolians had a significantly lower proportion of insufficient intake of vegetables and fruits than Han Chinese (80·6% vs· 82·2%), the association between GPL and vegetables and fruits consumption was more evident for Mongolians (P < 0·05). The results of this study contrast with some previous studies that claimed vitamins, minerals, and antioxidants from fruits and vegetables could prevent gastric cancer by modulating DNA methylation and inhibiting gastric cancer cell growth (34). We identified some factors that might serve to explain this inconsistency.

First, in addition to the differences in the consumption of fruits and vegetables, Mongolians and Han Chinese also differ in other aspects of their dietary structure, the most notable of which is their consumption of meat and dairy products. A recent investigation supported this claim, showing that urban Mongolian dwellers consume meat, milk, and dietary products more frequently than urban Han Chinese dwellers (35), an indicator of Mongolians’ nomadic heritage. However, such dietary habits might elevate Mongolians’ risk for GC. A previous Meta-analysis study suggested an increased risk associated with red or processed meat for GC (36). Moreover, dairy products have also been found to assume a significant positive correlation with the development of chronic atrophic gastritis and gastric cancer (37).

Second, genetic predisposition might also elevate the risk of GC among Mongolians. A recent study showed that the concentration of serum leptin, which is related to the progression and angiogenesis of gastric cancer and predicts poorer prognostic outcomes (38), was significantly higher in Mongolians than in Han Chinese(3·58 ± 1·85 ng/ml and 3·02 ± 1·75 ng/ml, respectively, P = 0·049) (39). It might seem counterintuitive since leptin levels are usually negatively correlated with fruits and vegetable consumption (40). Despite the lack of direct evidence, we hypothesize that genetic variations might exist between Mongolians and Han Chinese and explain the different levels of leptin in both groups.

(4) Our result also indicated that the proportion of smokers in Mongolians was higher than in Chinese Hans (23·1% vs 18·80%). However, the association between smoking and GPL was significantly lower among Mongolians (OR 2·96 vs 5·24), resulting in similar PAFs between the two groups. We believe several biological and social factors might be contributing to this phenomenon.

First, smoking might lower the risk of H· pylori infection, thus preventing the development of GC. A previous study has shown that current smokers have a lower risk of H· pylori infection than people who have never smoked (41). There is a possibility that smoking increases acid production and secretion of pepsin that protects the gastric mucosa against H· pylori infection (42).

Second, the lower GPL risk in Mongolians may be explained by a more diverse gut microbiome compared with the Han Chinese. The metagenomic species analysis indicated that lactose-digesting Bifidobacterium species were abundant in Mongolian people, which might contribute to gut health through their anti-inflammatory properties and butyrate production ability (43). An earlier study showed that ingestion of Bifidobacterium alone improved control of melanoma tumors to the same extent as PDL1-specific antibody therapy (44).

Third, despite Han Chinese reporting a lower smoking prevalence, the data might be biased by the negative social connotations of smoking (45). Due to the influence of Confucianism, 58·9% of Korean female smokers who were cotinine-verified classified themselves as nonsmokers in self-reports (46). Han Chinese, who share similar cultural backgrounds, are also likely to underreport smoking to evade possible negative judgment.

### Strengths

To our knowledge, the current study is likely the first to investigate the association between multiple lifestyle factors and the risk of GPL among major minority groups in the largest Mongolian residence in China. Besides, the result of this research could also be potentially applicable to Mongolian populations in other geographical areas and might be helpful in future comparative studies that investigate etiological differences in different Mongolian groups. Furthermore, the notable ethnic differences on the association between lifestyle factors and risk for GPL revealed in this study shed light on the potential role ethnicity plays in disease formation, which might facilitate the formation of health policies that cater to the specific needs of ethnic minorities. In addition, we included detailed information on the number of cigarettes consumed daily, which allowed us to estimate the dose-response relationship between smoking and GPL risk.

### Limitations

This study has several limitations. First, the cross-sectional design of our study makes it impossible to infer a causal relationship. Nevertheless, the study identified a range of etiological hypotheses regarding ethnic disparities, which could guide further studies. Second, as the trends in exposure to life style risk factors during the past three decades is not considered, the probable cohort effect cannot be estimated in this cross-sectional study. This study may also be subject to recall bias and length bias. But recall bias may be limited as information on lifestyle factors in the questionnaire were asked before any condition of interest. Moreover, such biases might lead to underestimated results because that undesirable habits such as smoking or drinking tend to be under-reported and unhealthy lifestyle factors were changed in prevalent GPL. Third, the present study cannot rule out the possibility of unmeasured confounders, including consumption of hot, spicy, and smoked foods, that might contribute to gastric cancer development. However, after the multivariable adjustment, most potential confounding effects would be controlled. Fourth, for physical activity, questionnaires to measure all activity domains (leisure time physical activity, activity at work, in the household, and for transport) were not adopted, so misclassification may be present in our study. Nevertheless, such measurement errors may not differentially affect subsequent health status and are likely to attenuate the relationship (16). In addition, we didn’t collect the type of alcoholic beverages consumed habitually and the amount of alcohol consumed on a typical drinking day to estimate the dose-response relationship between alcohol consumption and GPL risk.

## Conclusions

In summary, our study suggested that alcohol consumption, physical inactivity, and insufficient vegetable and fruit intake increased the risk of GPL among Mongolians to a greater extent than Han Chinese, reflecting a potential difference in GPL pathogenesis in these two groups. This present study provides crucial implications that high-risk lifestyle factors should be reduced, particularly in Mongolians. More information is needed on how lifestyle factors, other host-related and environmental risk factors cooperate in the oncogenic cascade across ethnic subgroups.

## Data Availability Statement

The raw data supporting the conclusions of this article will be made available by the authors, without undue reservation.

## Ethics Statement

The studies involving human participants were reviewed and approved by Ethics Committee of National Cancer Center/Cancer Hospital. The patients/participants provided their written informed consent to participate in this study.

## Author Contributions

WW, LQ, SW, and YX had the idea for the study and contributed to the study design. LQ, WD, JR, PD, and YX coordinated data acquisition and standardization. WD and SW analyzed data. WW, LQ, WD, JR, XC, SZ, SW, PD, and YX interpreted data. WW and XC wrote the draft report, and all authors contributed to the revision of the report. WW, LQ, SW, and YX have full access to all the data in the study. All authors critically interpreted the results and developed the report. All authors reviewed and approved the final version.

## Funding

Local science and technology development fund projects guided by the central government (2020ZY0015); Natural Science Foundation of Inner Mongolia Autonomous Region, China (2021MS08039); National Key Research and Development Program of China (2018YFC1311704).

## Conflict of Interest

The authors declare that the research was conducted in the absence of any commercial or financial relationships that could be construed as a potential conflict of interest.

## Publisher’s Note

All claims expressed in this article are solely those of the authors and do not necessarily represent those of their affiliated organizations, or those of the publisher, the editors and the reviewers. Any product that may be evaluated in this article, or claim that may be made by its manufacturer, is not guaranteed or endorsed by the publisher.
