# Supplementary material for: Differences in the Association Between Modifiable Lifestyle Factors and Gastric Precancerous Lesions Among Mongolians and Han Chinese
Source: Front Oncol. 2022 Jun 2;12:798829. doi: 10.3389/fonc.2022.798829 (PMC9200956; doi:10.3389/fonc.2022.798829)
Supplement: Supplementary file 1 [file Table_1.docx]

Supplementary Material

# Supplementary Table 1. Ranked proportions of all risk factors* and combinations of multiple behavioral risk factors

| Number of lifestyle factors | Current Smoking | Alcohol consumption | Physically inactive | Insufficient vegetables and fruits intake | n (%) |
| --- | --- | --- | --- | --- | --- |
| Four | Y | Y | Y | Y | 5175 (8.40) |
| Three | N | Y | Y | Y | 1553 (2.52) |
|  | Y | N | Y | Y | 2077 (3.37) |
|  | Y | Y | N | Y | 1689 (2.74) |
|  | Y | Y | Y | N | 755 (1.22) |
| Two | N | N | Y | Y | 24399 (39.58) |
|  | N | Y | N | Y | 1487 (2.41) |
|  | N | Y | Y | N | 314 (0.51) |
|  | Y | N | N | Y | 912 (1.48) |
|  | Y | N | Y | N | 367 (0.60) |
|  | Y | Y | N | N | 530 (0.86) |
| One | Y | N | N | N | 376 (0.61) |
|  | N | Y | N | N | 532 (0.86) |
|  | N | N | Y | N | 3613 (5.86) |
|  | N | N | N | Y | 13277 (21.54) |
| None | N | N | N | N | 4582 (7.43) |

Note: *Current smokers were defined as those who had smoked ≥ 1 cigarette (or equivalent) per day for at least 6 months at baseline. Alcohol consumption was defined as drinking alcohol at least once a week on average for more than 6 consecutive months. Regular physical activity was defined as exercise at least ≥30 minutes on 3 days of the week. Insufficient vegetables and fruits intake were defined as consuming less than 360g vegetables and 180g fruits per day on average.

**Supplementary Table 2. Multivariable-adjusted ORs(95%CIs) for GPL by lifestyle factors grouped by ethnicity**

|  | **Model 1**  (Age, Sex) | |  | **Model 2**  (Age, Sex, Education, Occupation) | |  | **Model 3**  (Age, Sex, Education, Occupation,  BMI, Family history of gastric cancer) | |
| --- | --- | --- | --- | --- | --- | --- | --- | --- |
|  | **OR (95%CI)** | ***P*-value**^*^ |  | **OR (95%CI)** | ***P*-value**^*^ |  | **OR (95%CI)** | ***P*-value**^*^ |
| **Age** |  | 0.068 |  |  | 0.101 |  |  | 0.010 |
| Han | 1.001 (0.998,1.004) |  |  | 1.00 (0.996,1.002) |  |  | 1.00(0.997,1.004) |  |
| Mongolians | 0.997 (0.989,1.005) |  |  | 1.00 (0.989, 1.005) |  |  | 0.997(0.998,1.007) |  |
| **Sex** |  | 0.036 |  |  | 0.029 |  |  | 0.050 |
| Han |  |  |  |  |  |  |  |  |
| Women | Ref |  |  | Ref |  |  | Ref |  |
| Men | 0.74 (0.70,0.79) |  |  | 0.73(0.69,0.77) |  |  | 0.72(0.68,0.77) |  |
| Mongolians |  |  |  |  |  |  |  |  |
| Women | Ref |  |  | Ref |  |  | Ref |  |
| Men | 0.88(0.76,1.01) |  |  | 0.86(0.75,1.00) |  |  | 0.86(0.73,1.00) |  |
| **Body mass index (kg/m^2^)** |  | 0.683 |  |  | 0.796 |  |  | 0.718 |
| Han |  |  |  |  |  |  |  |  |
| <18.5 | 1.08(0.88,1.33) |  |  | 1.15(0.93,1.43) |  |  | 1.29(1.03,1.62) ^*^ |  |
| 18.5-23.9 | Ref |  |  | Ref |  |  | Ref |  |
| 24-27.9 | 1.24(1.16,1.31) ^*^ |  |  | 1.23(1.16,1.31) ^*^ |  |  | 1.21(1.13,1.62) ^*^ |  |
| ≥28 | 1.57(1.42,1.74) ^*^ |  |  | 1.57(1.42,1.74) ^*^ |  |  | 1.59(1.42,1.78) ^*^ |  |
| Mongolians |  |  |  |  |  |  |  |  |
| <18.5 | 1.55 (0.92,2.60) |  |  | 1.58(0.93,2.68) |  |  | 1.64(0.93,2.87) |  |
| 18.5-23.9 | Ref |  |  | Ref |  |  | Ref |  |
| 24-27.9 | 1.23(1.05,1.43) ^*^ |  |  | 1.22(1.04,1.42) ^*^ |  |  | 1.14(0.97,1.35) |  |
| ≥28 | 1.66(1.33,2.07) ^*^ |  |  | 1.68(1.33,2.09) ^*^ |  |  | 1.50(1.18,2.87) ^*^ |  |
| **Occupation** |  | 0.271 |  |  | 0.317 |  |  | 0.508 |
| Han |  |  |  |  |  |  |  |  |
| Public officer | ref |  |  | ref |  |  | ref |  |
| Agricultural and industrial service personnel | 1.89(1.77,2.03) ^*^ |  |  | 1.72(1.61,1.85)^*^ |  |  | 1.52(1.41,1.65) ^*^ |  |
| House worker | 1.01(0.92,1.12) |  |  | 0.93(0.84,1.03) |  |  | 0.94(0.84,1.04) |  |
| Others | 1.36(1.17,1.59) ^*^ |  |  | 1.32(1.13,1.54)^*^ |  |  | 1.29(1.09,1.53) ^*^ |  |
| Mongolians |  |  |  |  |  |  |  |  |
| Public officer | ref |  |  | ref |  |  | ref |  |
| Agricultural and industrial service personnel | 1.95(1.95,2.30) ^*^ |  |  | 1.78(1.48,2.15)^*^ |  |  | 1.72(1.41,2.11) ^*^ |  |
| House worker | 1.25(0.99,1.58) |  |  | 1.13(0.88,1.45) |  |  | 1.13(0.86,1.48) |  |
| Others | 1.08(0.77,1.53) |  |  | 1.01(0.71,1.43) |  |  | 1.01(0.69,1.47) |  |

Note: Abbreviations: OR, odds ratio; CI, confidence interval. *P-value of interaction between Mongolians and Hans.

**Supplementary Table 3. Multivariable-adjusted ORs (95%CIs) for GPL by lifestyle factors^*^ grouped by ethnicity in individuals without family history of gastric cancer**

|  | **Model 1** (Age, Sex) | | **Model 2**  (Age, Sex, Education, Occupation) | | | **Model 3**  (Age, Sex, Education, Occupation,  BMI^†^, Family history of gastric cancer) | | |
| --- | --- | --- | --- | --- | --- | --- | --- | --- |
|  | **OR (95%CI)** | ***P*-value**^‡^ | **OR (95%CI)** | ***P*-value**^‡^ | **OR (95%CI)** | | ***P*-value**^‡^ |  |
| **Smoking^§^** |  | 0.007 |  | 0.003 |  | | 0.004 |  |
| Han |  |  |  |  |  | |  |  |
| Never | Ref |  | Ref |  | Ref | |  |  |
| <11 cigarettes/d | 6.31(5.62,7.10) |  | 6.13(5.45,6.90) |  | 5.99(5.32,6.74) | |  |  |
| 11-20 cigarettes/d | 9.48(8.64,10.4) |  | 9.14(8.33,10.03) |  | 8.94(8.14,9.82) | |  |  |
| ≥21 cigarettes/d | 6.94(6.24,7.73) |  | 6.59(5.91,7.35) |  | 6.51(5.84,7.26) | |  |  |
| Mongolians |  |  |  |  |  | |  |  |
| Never | Ref |  | Ref |  | Ref | |  |  |
| <11 cigarettes/d | 3.63(2.67,4.94) |  | 3.43(2.51,4.68) |  | 3.35(2.45,4.57) | |  |  |
| 11-20 cigarettes/d | 6.94(5.59,8.62) |  | 6.55(5.26,8.15) |  | 6.50(5.21,8.11) | |  |  |
| ≥21 cigarettes/d | 6.84(5.2,9.00) |  | 6.73(5.10,8.88) |  | 6.67(5.05,8.81) | |  |  |
| **Alcohol consumption** |  | 0.019 |  | 0.017 |  | | 0.016 |  |
| Han | 5.93(5.53,6.36) |  | 5.78(5.39,6.21) |  | 5.68(5.29,6.10) | |  |  |
| Mongolians | 7.34(6.14,8.77) |  | 7.09(5.92,8.48) |  | 7.03(5.87,8.43) | |  |  |
| **Physically inactive** |  | <0.001 |  | <0.001 |  | | <0.001 |  |
| Han | 1.08(1.01,1.15) |  | 1.03(0.96,1.10) |  | 1.04(0.97,1.11) | |  |  |
| Mongolians | 2.11(1.77,2.53) |  | 2.01(1.68,2.42) |  | 1.99(1.66,2.40) | |  |  |
| **Insufficient vegetables and fruits intake** |  | 0.065 |  | 0.062 |  | | 0.089 |  |
| Han | 1.02(0.94,1.11) |  | 0.98(0.90,1.07) |  | 1.02(0.94,1.12) | |  |  |
| Mongolians | 1.25(1.01,1.55) |  | 1.22(0.98,1.51) |  | 1.25(1.01,1.55) | |  |  |
| **Lifestyle risk factor index^¶^** |  | <0.001 |  | <0.001 |  | | 0.001 |  |
| Han |  |  |  |  |  | |  |  |
| 0 | Ref |  | Ref |  | Ref | |  |  |
| 1 | 0.75(0.64,0.87) |  | 0.73(0.63,0.85) |  | 0.75(0.65,0.87) | |  |  |
| 2 | 0.87(0.75,1.00) |  | 0.83(0.72,0.96) |  | 0.86(0.74,0.99) | |  |  |
| 3 | 3.89(3.34,4.53) |  | 3.64(3.13,4.25) |  | 3.67(3.14,4.28) | |  |  |
| 4 | 7.16(6.15,8.34) |  | 6.62(5.68,7.71) |  | 6.70(5.75,7.81) | |  |  |
| Mongolians |  |  |  |  |  | |  |  |
| 0 | Ref |  | Ref |  | Ref | |  |  |
| 1 | 0.66(0.44,0.98) |  | 0.64(0.43,0.96) |  | 0.65(0.44,0.97) | |  |  |
| 2 | 1.21(0.84,1.74) |  | 1.18(0.81,1.70) |  | 1.19(0.82,1.72) | |  |  |
| 3 | 3.77(2.56,5.55) |  | 3.62(2.45,5.36) |  | 3.60(2.44,5.33) | |  |  |
| 4 | 10.15(6.94,14.85) |  | 9.42(6.42,13.84) |  | 9.37(6.37,13.77) | |  |  |

Note: Abbreviations: OR, odds ratio; CI, confidence interval.

**^*^**Smokers were defined as those who had smoked ≥ 1 cigarette (or equivalent) per day for at least 6 months. Alcohol consumption was defined as drinking alcohol at least once a week on average for more than 6 consecutive months. Regular physical activity was defined as exercise at least ≥30 minutes on 3 days of the week. Insufficient vegetables and fruits intake were defined as consuming less than 360g vegetables and 180g fruits per day on average. ^§^158 (0.26%) missing. ^‡^P-value of interaction between Mongolians and Han*.* ^†^ 325 (0.53%) missing. ^¶^Lifestyle risk factor index was calculated by summing the individual scores of four risk factors (1=yes, 0=no): smoking, drinking, physically inactive, and insufficient intake of vegetables and fruits.

**Supplementary Table 4. Multivariable-adjusted ORs (95%CIs) for GPL by smoking and drinking*grouped by sex and ethnicity**

| **Category** | **OR^†^ (95% CI)** | ***P-value^‡^*** |
| --- | --- | --- |
| Sex |  |  |
| Men |  | 0.2381 |
| Han |  |  |
| No smoking & No drinking | Ref |  |
| Smoking or Drinking | 6.45(5.58,7.46) |  |
| Smoking & Drinking | 13.31(11.71,15.14) |  |
| Mongolians |  |  |
| No smoking & No drinking | Ref |  |
| Smoking or Drinking | 6.53(4.23,10.10) |  |
| Smoking & Drinking | 17.29(11.59,25.8) |  |
| Women |  | 0.0396 |
| Han |  |  |
| No smoking & No drinking | Ref |  |
| Smoking or Drinking | 3.82(3.42,4.28) |  |
| Smoking & Drinking | 7.32(6.55,8.18) |  |
| Mongolians |  |  |
| No smoking & No drinking | Ref |  |
| Smoking or Drinking | 3.05(2.35,3.94) |  |
| Smoking & Drinking | 8.60(6.66,11.09) |  |

Note: OR, odds ratio; CI, confidence interval. *Smokers were defined as those who had smoked ≥ 1 cigarette (or equivalent) per day for at least 6 months. Alcohol consumption was defined as drinking alcohol at least once a week on average for more than 6 consecutive months. †Odds ratio was adjusted for age, education, occupation, body mass index, family history of gastric cancer. ‡P-value of interaction between sex and ethnicity.
